# Establishing a unique specimen repository with characterized G6PD enzyme activity for the development of new G6PD point-of-care tests

**DOI:** 10.1186/1475-2875-13-S1-O12

**Published:** 2014-09-22

**Authors:** Maria Kahn, Nicole LaRue, Jeffrey Wellhausen, Sampa Pal, Gonzalo J Domingo

**Affiliations:** 1 PATH, Diagnostic Technologies, Seattle, WA, USA

## 

Glucose-6-phosphate dehydrogenase (G6PD) deficiency tests play a critical role in supporting the safe treatment and elimination of malaria. Evaluating G6PD activity in those infected with *Plasmodium vivax* will be essential in order to treat and achieve radical cures. Our goal is to establish a unique specimen repository with cryopreserved specimens highly characterized for G6PD activity. Providing such specimens to designers of G6PD point-of-care tests, will support both the development and evaluation of new diagnostic devices. Because G6PD-deficiency prevalence ranges from <1–>15%, obtaining fresh blood specimens with a range of levels of G6PD activity - as well as specimens from heterozygous females - typically requires constant screening from large numbers of subjects. Thus, developers would highly benefit by using the same panel of specimens from the repository over the entire course of the development of a specific G6PD test. Currently the repository constitutes specimens from adults of known G6PD-deficient population groups and will be expanded over time with diverse ethnic backgrounds. Standard operation procedures and formulations have been optimized to allow cryopreservation of red blood cells (RBC) and stabilize G6PD enzyme activity within the RBC. Flow cytometry and kinetic assays demonstrate that specimens can be stored a minimum of six months under these optimized conditions. Critically, thawed specimens demonstrate stability for seven days. We are using Freezerworks database to maintain the chain of custody of all specimens. Cytochemical staining is performed to pedigree the specimens. The specimens are processed and tested for G6PD activity immediately by quantitative and qualitative assays. Samples are stored depending on the phenotype in order to increase the capacity of G6PD diverse specimens. Samples with normal G6PD activity are more prevalent and discarded to accommodate intermediate and deficient specimens. Once samples have been tested and selected for the repository, three preservation methods are used: (1) whole RBCs are cryopreserved in glycerolyte and stored in liquid nitrogen for G6PD phenotyping, (2) whole RBCs without glycerolyte are preserved in liquid nitrogen for phenotyping, and (3) peripheral mononuclear blood cells are preserved for DNA sequencing and genotyping. The G6PD specimen repository at PATH will provide a low-cost service to research, commercial, and other organizations with compatible product development goals that benefit the global health community.

